# Supplementary material for: A Critical View of the Application of the APEX Software (Aqueous Photochemistry of Environmentally-Occurring Xenobiotics) to Predict Photoreaction Kinetics in Surface Freshwaters
Source: Molecules. 2019 Dec 18;25(1):9. doi: 10.3390/molecules25010009 (PMC7017383; doi:10.3390/molecules25010009)
Supplement: Supplementary file 1 [file molecules-25-00009-s001.zip › 3.2.4_gcc-4.4.0.zip!/3.2.4_gcc-4.4.0/3.2.4_gcc-4.4.0/doc/octave/html/interpreter/Precedence-of-Objects.html]

Precedence of Objects - Untitled


Previous: Operator Overloading,
Up: Overloading Objects


---

#### 33.4.3 Precedence of Objects

Many functions and operators take two or more arguments and so the
case can easily arise that these functions are called with objects of
different classes. It is therefore necessary to determine the precedence
of which method of which class to call when there are mixed objects given
to a function or operator. To do this the `superiorto` and
`inferiorto` functions can be used

— Built-in Function: **superiorto** (class\_name, ...)  
> When called from a class constructor, mark the object currently
> constructed as having a higher precedence than class\_name.
> More that one such class can be specified in a single call.
> This function may only be called from a class constructor.

— Built-in Function: **inferiorto** (class\_name, ...)  
> When called from a class constructor, mark the object currently
> constructed as having a lower precedence than class\_name.
> More that one such class can be specified in a single call.
> This function may only be called from a class constructor.

For example with our polynomial class consider the case

```
     2 * polynomial ([1, 0, 1]);
```

That mixes an object of the class "double" with an object of the class
"polynomial". In this case we like to ensure that the return type of
the above is of the type "polynomial" and so we use the
`superiorto` function in the class constructor. In particular our
polynomial class constructor would be modified to be

```
```
     ## -*- texinfo -*-
     ## @deftypefn {Function File} {} polynomial ()
     ## @deftypefnx {Function File} {} polynomial (@var{a})
     ## Creates a polynomial object representing the polynomial
     ##
     ## @example
     ## a0 + a1 * x + a2 * x^2 + @dots{} + an * x^n
     ## @end example
     ##
     ## from a vector of coefficients [a0 a1 a2 ... an].
     ## @end deftypefn
     
     function p = polynomial (a)
       if (nargin == 0)
         p.poly = [0];
         p = class (p, "polynomial");
       elseif (nargin == 1)
         if (strcmp (class (a), "polynomial"))
           p = a;
         elseif (isvector (a) && isreal (a))
           p.poly = a(:).';
           p = class (p, "polynomial");
         else
           error ("polynomial: expecting real vector");
         endif
       else
         print_usage ();
       endif
       superiorto ("double");
     endfunction
```
```

Note that user classes always have higher precedence than built-in
Octave types. So in fact marking our polynomial class higher than the
"double" class is in fact not necessary.
